# Hemiplegia

**Published:** 1901-10-26

**Authors:** 


### HEMIPLEGIA.

When a patient is attacked with hemiplegia he is likely before long to suffer from many difficulties besides the mere deprivation of control over his muscular apparatus. Dr. Leonard Guthrie[1] drawing attention to these secondary consequences of hemiplegia, and indicating their treatment, says that in the more extreme condition of helplessness into which the paralytic may drift, the immobility of the limbs is due (1) to articular adhesions; (2) to motor paralysis leading to muscular atrophy; and (3) to spasticity or spasmodic contraction of muscles, leading to permanent shortening of the stronger ones. Articular adhesions frequently cause limitations of movement. They are most common, and are formed earliest in the shoulder, rendering movements in the joint painful if not impossible. These adhesions often render a limb useless which might have recovered but for their presence, but, whatever be their cause, in most cases they are preventible. Unfortunately it happens too often that in the early days of hemiplegia a dread is entertained lest any disturbance of the patient should cause further brain mischief. This fear of moving the limbs is quite unfounded, and gentle passive movement should be practised many times a day from the very first, for adhesions begin to form very early, in the first week or two, and when formed they are very difficult to disperse. Spasticity or early rigidity may occur in any lesion affecting the cerebral motor tract. Early treatment is important. Almost from the first the limbs tend to assume the attitude which may afterwards become permanent, therefore, even whilst the patient is confined to bed, all faulty positions and any tendency to adopt a particular position should be corrected. Muscular atrophy usually occurs somewhat later in hemiplegic limbs. It is doubtless due to disuse, but it must be remembered that nervous, as well as muscular tissues may suffer from this cause, and if normal stimuli are absent, artificial stimuli such as passive movements, massage and electricity must take their place. By them we may hope to preserve the nutrition of muscles and neurons alike. The means at our disposal in the treatment of these various results of hemiplegia are passive movements, massage and electricity, re-education of movements by passive and active exercises combined, and mechano-therapeutics. In the application of massage, the general rule should be to massage the opponents of the contracting muscles, the contracting muscles being left alone. Electricity, whether Faradism or galvanism is a useful adjunct to massage, but cannot take its place. Strong currents are to be avoided. Re-education of movements is to be obtained by the movements being gone through passively at first, and then by degrees actively. It is useless merely to tell a patient to elevate his arm at the shoulder. He will only sigh, "I cannot." But tell him to do so, and at the same time do it for him, and he will gradually learn to do it for himself. At each attempt the operator does less, and the patient more. This principle may be applied in the re-education and amplification of all movements. In all attempts at re-education, movements should be first encouraged in those parts which naturally recover first. It is important to find out what a patient can do, and to make him do it. Mechano-therapeutics by weights, pulleys, etc., are only of use to increase powers already partially regained.

[1] Lancet, Oct. 19.

